# Muscle membrane repair and inflammatory attack in dysferlinopathy

**DOI:** 10.1186/2044-5040-1-10

**Published:** 2011-03-01

**Authors:** Renzhi Han

**Affiliations:** 1 Department of Cell and Molecular Physiology, Stritch School of Medicine, Loyola University Medical Center, Maywood, IL 60153, USA

## Abstract

Repair of plasma membrane tears is an important normal physiological process that enables the cells to survive a variety of physiological and pathological membrane lesions. Dysferlin was the first protein reported to play a crucial role in this repair process in muscle, and recently, several other proteins including Mitsugumin 53 (MG53), annexin and calpain were also found to participate. These findings have now established the framework of the membrane repair mechanism. Defective membrane repair in dysferlin-deficient muscle leads to the development of muscular dystrophy associated with remarkable muscle inflammation. Recent studies have demonstrated a crosstalk between defective membrane repair and immunological attack, thus unveiling a new pathophysiological mechanism of dysferlinopathy. Here I summarize and discuss the latest progress in the molecular mechanisms of membrane repair and the pathogenesis of dysferlinopathy. Discussion about potential therapeutic applications of these findings is also provided.

## Introduction

Damage to the plasma membrane induces entry of toxic agents such as calcium and oxidants into the cells, releases intracellular molecules producing inflammatory responses, and threatens the afflicted cells with an immediate cell death. Recent studies reveal a rapid membrane repair response that is conserved in many different types of cells to restore the plasma membrane integrity and enables the cells to survive following a limited level of membrane disruptions [1-7]. Defects in this process can result in pathological complications in a number of different tissues, particularly the skeletal muscle and heart [8-11]. Moreover, continuous release of intracellular contents from cells with defective membrane repair exposes "danger" signals to the immune system of the host and causes further tissue damage [12-14].

## Molecular mechanism underlying muscle membrane repair

**Figure 1:**
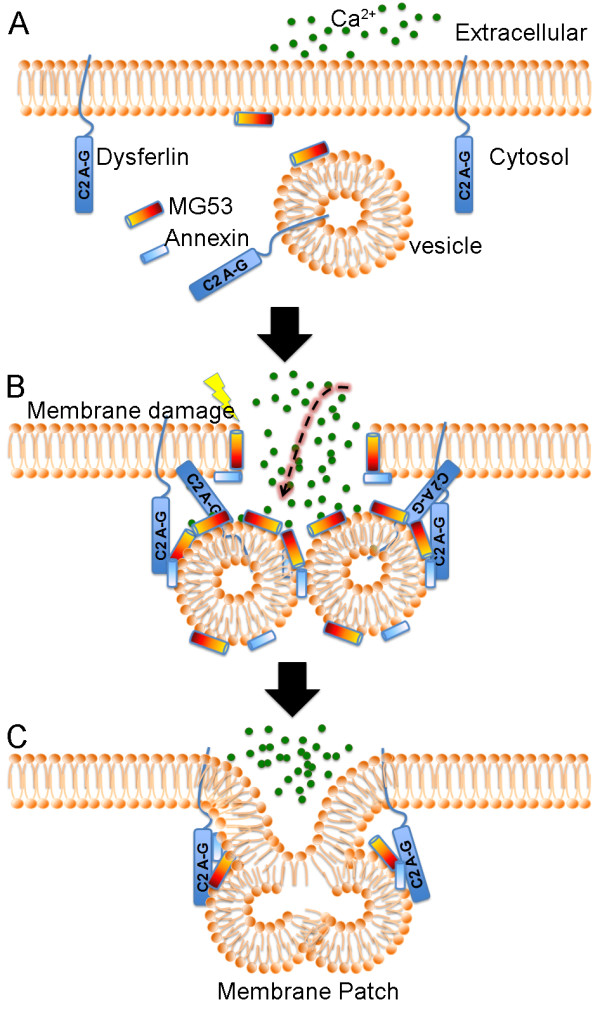
**A schematic model for muscle membrane repair**. (A) In uninjured muscle, the intact sarcolemma separates the intracellular environment from the hostile extracellular environment. (B) Membrane disruption leads to Ca^2+ ^influx, oxidant entry and cholesterol unfurling. Membrane repair vesicles are transported towards the damage site by the motor proteins kinesin and myosin, and this process may be facilitated by Mitsugumin 53 (MG53) in the oxidation/cholesterol-dependent manner. The vesicles dock via oxidized MG53 and fuse with each other and with the plasma membrane possibly mediated by annexin, SNAREs and dysferlin in the presence of Ca^2+^. (C) A membrane "patch" is consequently formed, which resealed the membrane lesion.

It is known that the membrane repair process requires intracellular vesicles [15] which deliver excess membrane to form a "membrane patch" through Ca^2+^-triggered vesicular exocytosis [16,17] similar to neurotransmitter release [18] (Figure 1). The intracellular vesicles are initially transported to the damage site via the sequential actions of the motor proteins including kinesin and non-muscle myosin IIA and IIB in sea urchin eggs and several cell lines such as 3T3 fibroblasts and COS-7 [19,20]. Myosin IIB is required for the exocytosis and membrane repair itself while myosin IIA is required in facilitation of cell membrane repair at repeated wounds [20]. However, the involvement of these motor proteins in muscle membrane repair has not been determined. Recently, Mitsugumin 53 (MG53), a muscle-specific tripartite motif family protein (TRIM72), has also come into play in vesicle translocation during muscle membrane repair [21-26]. MG53 is observed to rapidly accumulate at the damage site following membrane disruption. Genetic ablation of MG53 results in a late-onset progressive skeletal myopathy [24] and increases susceptibility to ischemia/reperfusion-induced myocardial damage [25,26]. Single myocytes isolated from MG53-deficient mice failed to reseal membrane disruptions created by laser irradiation, focal electroporation or microneedle penetration [24-26]. Consistent with the role of MG53 to recruit vesicles during membrane repair, electron microscopy examination of MG53-deficient muscle fibers observed membrane breaks without accumulation of vesicles at the damage site [24]. These studies have suggested that MG53 plays a role in facilitating vesicle translocation for muscle membrane repair. Interestingly, the translocation of MG53 upon membrane damage is Ca^2+^-independent, but rather mediated by cholesterol exposure and oxidation-induced oligomerization in skeletal and cardiac muscle [24,25]. This suggests that MG53-mediated vesicle translocation and Ca^2+^-triggered vesicle-membrane fusion are two distinct steps in the membrane resealing process. Thus, Ca^2+^, cholesterol and oxidation can trigger different components of the membrane repair machinery to initiate the emergency response. It is interesting what other signals could be involved in the initiation of the membrane repair responses. In addition to Ca^2+ ^influx, cholesterol exposure and oxidation, membrane damage can result in other changes such as Na^+ ^influx and membrane potential depolarization, which may also be involved in membrane repair.

After vesicle translocation, Ca^2+^-regulated fusion of the vesicles with the plasma membrane is followed to form a "membrane patch". Intracellular vesicle fusion generally involves the SNARE (soluble N-ethylmaleimide-sensitive factor attachment protein receptors) proteins [27] and synaptotagmins (Syt), transmembrane proteins with two highly conserved C2 domains that may serve as calcium sensors in the regulation of vesicle exocytosis in neurons and other cell types [28]. Previous studies have demonstrated a potential role of SNARE proteins in membrane resealing [18,29-31]. Syt7 was shown to be involved in membrane repair of fibroblasts [32]. Genetic ablation of Syt7 in mice resulted in inflammatory myopathy with extensive fibrosis, high serum creatine kinase levels and progressive muscle weakness [33]. Moreover, recent studies reported that Syt1 participates in Ca^2+^-dependent repair of membranes in plants [34,35]. These studies highlight a conservation of membrane repair mechanisms between animal and plant cells. Structurally similar to Syt, dysferlin contains multiple C2 domains and shows Ca^2+^-sensitive phospholipid binding activities [36,37]. Dysferlin has been well established as an important player for muscle membrane repair although the underlying mechanism remains poorly understood. It is possible that dysferlin functions as a Ca^2+ ^sensor and directly regulates the SNARE-mediated vesicle-membrane fusion during membrane repair. Direct interactions between dysferlin and SNARE proteins have not been established yet. However, otoferlin, a mammalian homologue of dysferlin, has been demonstrated to bind synaptosomal-associated protein 25 (SNAP25) and syntaxin-1 and directly regulate synaptic vesicle exocytosis in inner hair cells [38,39]. Thus, establishing if dysferlin interacts with SNARE proteins and can influence the SNARE-mediated membrane fusion is of potential interest in future studies. In addition to SNARE-mediated membrane fusion, other fusogens including annexin and phospholipase A_2 _(PLA_2_) may also regulate the membrane repair process. Annexin A1 was shown to concentrate at the site of membrane damage and ablation of annexin A1 effectively inhibits membrane repair [40]. The activation of PLA_2 _and the generation of arachidonic acid promote membrane fusion mediating neutrophil degranulation [41]. Moreover, membrane sealing at the cut end of the giant axon has been shown to involve the activation of PLA_2 _[42,43]. Whether these fusogens are involved in membrane repair of skeletal muscle remains to be explored. The dysferlin-interacting protein AHNAK [44] was shown to bind phospholipase C in the presence of arachidonic acid [45,46]. This might provide a link between dysferlin and fusogens.

Except for dysferlin, Syt7 and MG53, the current knowledge regarding membrane repair is largely from non-muscle studies. Future studies are necessary to bridge our knowledge gap in skeletal muscle membrane repair. Since membrane repair is highly conserved during evolution, it is no wonder that skeletal muscle may use some of the common mechanisms as identified in other cells to repair membrane damage. However, skeletal muscle cells are quite special in that they undergo frequent mechanical stress, which causes frequent membrane disruption and thus may require skeletal muscle cells to use some unique mechanisms to satisfy the high demand of membrane repair.

## New functions of dysferlin

Recent studies have revealed new functions of dysferlin which may be linked to membrane repair and/or inflammatory activation. Dysferlin was observed to associate with developing T-tubules [47,48] and interact with dihydropyridine receptor (DHPR) [49]. Ultrastructural analysis of dysferlin-deficient muscle revealed primary T-tubule abnormalities similar to those seen in caveolin-3-deficient muscle [48], suggesting that dysferlin is required for correct T-tubule formation and/or maintenance. It is intriguing to examine whether the T-tubule is involved in muscle membrane repair and whether the T-tubule defect in dysferlin-deficient muscle underlies the compromised membrane repair.

Dysferlin may coordinate cytoskeleton remodeling through its interaction with focal adhesion components. Previous work showed that dysferlin interacts with β-parvin [50], a protein that directly interacts with integrin linked kinase and is important for stabilizing focal adhesions [51,52]. Such an interaction was further confirmed in a recent study using proteomic analysis of the dysferlin protein complex [53]. In this latter study, several other focal adhesion molecules including vinculin, actinin and talin were also identified in the dysferlin protein complex. In addition, dysferlin was reported to interact with α-tubulin [54]. These data suggest a role of dysferlin in cytoskeleton remodeling and focal adhesion, which have been proposed to facilitate vesicle trafficking and fusion during membrane repair [55,56].

Recent data found that dysferlin may also be involved in cytokine and/or chemokine secretion. Cultured myoblasts from dysferlin mutant mice showed impaired secretion of cytokine MCP-1 when stimulated with IFN-γ or damaged with saponin [57]. The authors of this study proposed that the impaired secretion of cytokines/chemokines in dysferlin-deficient muscle may account for the delayed neutrophil recruitment and thus the attenuated muscle regeneration [57]. A more recent study reported that dysferlin-deficient myoblasts and myotubes from human patients released more soluble factors involved in monocyte chemotaxis than control cells [58]. It is unclear whether this discrepancy is due to different species or other reasons (for example, different stages of disease). The potential role of dysferlin in the release of cytokines and other inflammatory mediators should be explored in the future studies. Recently, it was found that dysferlin is involved in the ATP release and Ca^2+^-triggered intercellular signaling in response to membrane wounding in fertilized sea urchin embryo [59]. Disruption of dysferlin expression by antisense morpholino in sea urchin embryo did not compromise the plasma membrane repair but effectively blocked the ATP release upon membrane damage and the consequent intercellular Ca^2+ ^signaling [59]. Interestingly, a recent study showed that skeletal muscle is capable of releasing IL-1β in response to combined treatment with lipopolysaccharide and the P2X7 receptor agonist, benzylated ATP [13], implicating the involvement of ATP signaling in muscle inflammation of dysferlinopathy. In contrast to the case with sea urchin embryo where disruption of dysferlin blocked the ATP release upon membrane damage, the authors proposed that dysferlin deficiency in mammalian skeletal muscle results in the ATP release possibly through a compensatory vesicle trafficking pathway mediated by synaptotagmin-like protein Slp2a and the small GTPase Rab27A, which activates the inflammasome pathway [13]. Future study to directly measure ATP release from dysferlin-deficient and control skeletal muscle in response to membrane damage should be conducted to validate this hypothesis. Additionally, ATP can be released from necrotic muscle fibers.

Finally, dysferlin was found to play a role in endothelial cell adhesion and angiogenesis [60,61]. Expression of dysferlin was observed in endothelial cells [61] and leaky brain blood vessels in multiple sclerosis [60]. Furthermore, dysferlin-deficient mice showed an impaired angiogenic response compared with control animals with angiogenic challenge [61], supporting an active role for dysferlin in endothelial homeostasis. How this defect in dysferlin-deficient subjects contributes to the pathogenesis of skeletal muscle is of potential interest and warrants future investigation.

## Crosstalk between defective membrane repair and immunological attack

**Figure 2:**
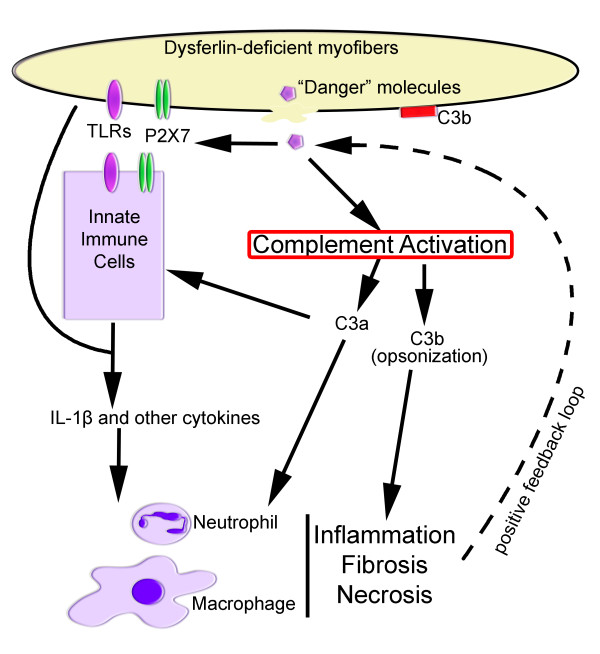
**Defective membrane repair and muscle inflammation**. Plasma membrane damage to dysferlin-deficient muscle fibers with compromised membrane repair causes a prolonged release of "danger" molecules, such as heat shock proteins (HSPs), high mobility group box 1 (HMGB1), ATP and uric acids. These "danger" molecules are recognized by receptors on leukocytes and the muscle fibers, stimulating generation of proinflammatory cytokines such as IL-1β. Other molecules that are exposed or released from the damaged cells activate the complement system, followed by the generation of proinflammation mediators (for example C3a, C5a) and opsonizing C3b. The proinflammatory mediators can trigger the production of proinflammatory cytokines from host cells and make the local vascular endothelium "leaky", thus attracting migration of neutrophils and monocytes. C3b binds to the negatively-charged sarcolemma, stimulating phagocytosis. These molecular and cellular responses cause more severe muscle damage and necrosis, leading to further release of the "danger" molecules and extensive muscle inflammation. The complement system and the inflammatory signaling pathway thus become attractive therapeutic targets for the treatment of dysferlinopathy.

It has been known that the immune system is able to produce remarkable responses in the absence of infectious organisms. A "danger" hypothesis has been put forward to explain how the immunological responses occur in these situations [12,13,62,63] (Figure 2). In this hypothesis, non-physiological cell death, damage or stress of the host cells, similar to those from the infectious organisms, can expose "danger" signals to the immune system [12,63,64]. The exact mechanism by which injured host cells influence the immunological responses is not fully understood. Some clues come from the observations showing that the injured cells induce dendritic-cell maturation [65], migration to draining lymph nodes *in vivo *[66], and complement activation [67]. Thus, it appears that the injured host cells can release endogenous adjuvants contributing to the initiation of the immunological responses [64].

Since plasma membrane integrity is important for preventing the release of endogenous molecules, a defective membrane repair in animal cells is inevitably immunologically dangerous. Several proteins including Syt7 [32,33], dysferlin [8,9,68], MG53 [22,24,25], and annexin A1 [40,68] have been shown to play a role in the membrane repair process. Accumulating evidence suggests a correlation between defective membrane repair and the development of abnormal inflammatory responses. For instance, genetic ablation of Syt7 in mice leads to the development of pathological alterations in the skin and skeletal muscle with many similarities to the polymyositis/dermatomyositis diseases in humans [33]. Disruption of annexin A1 also leads to complications in the inflammatory responses although the inflammatory responses in annexin A1-null mice have not been attributed to its role in membrane repair [69,70]. Dysferlinopathy is well known for the presence of a prominent muscle inflammation [71-73], and some of the dysferlinopathy patients were even initially misdiagnosed as having polymyositis [74,75]. Moreover, although highly resistant to lengthening-contraction-induced injury [14,57], dysferlin-deficient muscle experienced a strong inflammatory response that delayed its recovery from injury caused by lengthening contractions [76,77].

Recent studies have begun to unveil the pathophysiological mechanisms underlying muscle inflammation in dysferlinopathy. Dysferlin-deficient monocytes from SJL/J mice and dysferlinopathy patients were reported to have increased phagocytic activity [78] and dysferlin deficiency induces an upregulation of inflammasome [13]. Disruption of dysferlin expression by RNA interference in the J774 macrophage cell line also significantly enhanced the phagocytosis, suggesting that the phagocytic defect in dysferlin-deficient monocytes is likely a direct consequence of dysferlin deficiency rather than a secondary effect due to the muscle pathology *in vivo *[78]. However, Chiu et al. in a recent paper [57] commented that the phagocytic activity of dysferlin-deficient monocytes from C57BL/10-SJL.Dysf mice that have a more controlled genetic background was not different from that of control cells. The reason underlying this discrepancy is not known. Comparison of the phagocytic activities of the monocytes from pre-pathological and post-pathological dysferlin mutant animals might provide some clues for this issue. Recent studies showed that muscle-specific transgenic expression of dysferlin at appropriate levels rescues the dystrophic phenotype in dysferlin mutant mice [14,79], suggesting that the enhanced phagocytic activity alone in dysferlin-deficient monocytes is not sufficient to cause muscle damage. Conditional knockout mice with specific disruption of dysferlin in the monocytes will be beneficial to further clarify this issue.

Previously, dysferlin-deficient muscle cells were reported to be susceptible to complement attack, which was attributed to the down-regulation of the complement regulator CD55 on the plasma membrane without dysferlin [80]. Activation of the complement system has been observed in dysferlin-deficient muscles from both the mice and humans [14,80,81]. The complement system, mainly composed of a number of circulating proteins as inactive precursors, is an important part of the innate immune system [82,83] and is involved in the development of inflammatory diseases [84]. In addition to protecting the hosts from the invasion of infectious organisms, the complement system can also contribute to the host tissue damage. This is evident in animal models of autoimmune diseases, such as glomerulonephritis, hemolytic anemia, myasthenia gravis, and in two nonimmunologically mediated forms of primary tissue damage, burn and ischemia (for a review, see [85]). Our recent study revealed that activation of the complement system plays an active role in the pathogenesis of dysferlinopathy [14]. Up-regulation of the complement factors in dysferlin-deficient muscles was observed in mice before the onset of the obvious pathological hallmarks [14]. Such an increased expression of the complement factors was normalized by muscle-specific expression of a dysferlin transgene [14], which also rescues the muscle pathology observed normally in the dysferlin mutant mice [14,79]. A genetic approach using complement-deficient mice with the disrupted expression of C3, a central component of the complement system, further confirmed the active role of complement activation in the progression of muscular dystrophy in dysferlin-deficient mice [14]. The terminal activation of the complement system produces the membrane attack complex (MAC) which forms a large pore on the plasma membrane and causes cell lysis. However, surprisingly we found that genetic ablation of the terminal component (C5) of the complement system had minimal effect on muscle pathology in dysferlin-deficient mice [14]. These results suggest that the activated C3 is responsible for the muscle damage in dysferlinopathy. Upon activation, C3 is cleaved into C3a and C3b. C3a is an anaphylotoxin that produces a local inflammatory response, and C3b serves as an opsonizing agent by coating the sarcolemma of dysferlin deficient muscle. Opsonization of the sarcolemma enhances the phagocytosis of the target cell by macrophages that are the predominant infiltrating cells in dysferlin-deficient muscles [73], either with or without C5. Thus, it is possible that C5 deficiency has minimal effect in dysferlinopathy. Additionally, nucleated cells are normally able to eliminate the cytolytic MAC from the plasma-membrane via Ca^2+^-dependent endocytic and exocytic processes [86].

Thus, the compromised membrane repair in the absence of dysferlin results in the prolonged release of endogenous "danger" molecules which lead to the local activation of the complement system [14] and upregulation of the inflammasome [13]. These "danger" molecules are recognized by receptors on leukocytes and the muscle fibers, stimulating the generation of proinflammatory cytokines such as IL-1β. Activation of the complement system generates proinflammatory mediators (for example, C3a, C5a) and opsonizing C3b. The proinflammatory mediators can trigger the production of proinflammatory cytokines from host cells and make the local vascular endothelium "leaky", thus attracting migration of neutrophils and monocytes. These molecular and cellular responses cause more severe muscle damage and necrosis, leading to further release of the "danger" molecules and extensive muscle inflammation. In support of this notion, it has been reported that the dysferlin-deficient muscle cells release more soluble factors than control cells [58]. Damaged cells are known to expose a number of "danger" molecules, such as heat shock proteins (HSPs) [87,88], uric acid [89], ATP [59], and the high mobility group box 1 (HMGB1) [90]. These molecules bind to their cellular receptors (Toll-like receptors (TLRs) and P2X7), activating inflammasome [13], nuclear factor κB (NF-κB) and the complement pathways [14] (Figure 2). The HSPs can induce complement activation in both an antibody-dependent and -independent manner without the presence of pathogens [67]. Interestingly, HSP70 was observed to be rapidly released into the circulation after acute myocardial infarction with the peak concentration correlated with creatine kinase, troponin T, IL-6 and IL-8 in the serum [91]. HMGB1, which is released by necrotic or damaged cells and secreted by activated monocytes and macrophages, also potently induces complement activation and inflammation [90]. HMGB1 was observed extranuclearly in muscle biopsies from patients with idiopathic inflammatory myopathies, and exposure of the isolated skeletal muscle to HMGB1 caused an irreversible decrease in Ca^2+ ^release from the sarcoplasmic reticulum [92]. The identity of the molecules released from the dysferlin-deficient muscle cells which can activate the complement system remains to be determined. It is interesting to explore whether HSPs, uric acid and HMGB1 are elevated in the serum of dysferlinopathy patients and animals. Disruption of these molecules (for example by RNA interference, mutant mouse models) should also be explored for their potentials in alleviating the muscle pathology in dysferlin-deficient mice.

## Therapeutic perspectives

Currently, there is no effective therapeutic treatment for dysferlinopathy patients. The primary defect lies in the defective membrane repair caused by dysferlin deficiency. Thus, gene replacement therapy to restore the expression of functional dysferlin and membrane repair represents a great promise. However, it is still far away from any clinical application in dysferlinopathy. Pharmacological interventions implicated from recent studies represent novel avenues for the treatment of this disease. Although the efficacy of anti-inflammatory corticosteroids in dysferlinopathy is still controversial [93], our recent finding using gene targeted mice demonstrated that targeting the complement system could be a therapeutic approach for dysferlinopathy [14]. Complement inhibition has already been explored as a therapeutic approach for the treatment of certain conditions involving excessive complement attack [94,95]. Other approaches to counteract the initiation of inflammation are also under consideration for the treatment of dysferlinopathy. For example, a recent study demonstrated that two dysferlinopathy patients treated with four weekly infusions of rituximab to deplete B cells improved the muscle strength in these patients [96]. Future studies of the signaling pathways to mitigate the inflammatory responses in dysferlinopathy should shed new lights into the design of pharmacological therapeutic strategies for the treatment of this disease.

## Conclusions

A protective membrane resealing mechanism at the cell level is highly conserved among different species and cell types. It is mediated by exocytosis of intracellular vesicles forming a membrane "patch" at the disruption site. This process requires the participation and coordination of a large group of proteins involving cytoskeleton remodeling, vesicle translocation, and membrane fusion. Some of these proteins have recently been discovered and linked to human diseases, emphasizing the importance of the membrane repair proteins in life. An acute membrane repair mechanism not only prevents damaged cells from necrosis, but also reduces the exposure of "danger" signals to the immune system, which otherwise amplify the signals and cause massive tissue injury.

## Competing interests

The authors declare that they have no competing interests.
